# Stoma Leakage: Prevalence, Associated Factors, and Assessment Tools—A Scoping Review

**DOI:** 10.3390/nursrep16020046

**Published:** 2026-01-30

**Authors:** Andrea Poliani, Ilaria Marcomini, Pietro Butti, Elena Dumitrita Nedesca, Duilio Fiorenzo Manara, Giulia Villa

**Affiliations:** Center for Nursing Research and Innovation, Faculty of Medicine and Surgery, Vita-Salute San Raffaele University, 20132 Milano, Italy; poliani.andrea@unisr.it (A.P.); butti.pie@gmail.com (P.B.); e.nedesca@studenti.unisr.it (E.D.N.); manara.duilio@hsr.it (D.F.M.); villa.giulia@hsr.it (G.V.)

**Keywords:** leakage, ostomy, prevalence, risk factors, scoping review, surgical stoma

## Abstract

**Background**: Peristomal leakage is one of the most troublesome complications of living with a stoma, affecting skin integrity, quality of life, and healthcare costs. However, definitions, measurement methods, and prevalence estimates remain heterogeneous. This scoping review aimed to (i) map the international prevalence of peristomal leakage across stoma subtypes; (ii) identify associated or correlated factors; and (iii) describe the tools used to assess leakage. **Methods**: A scoping review was performed following the Joanna Briggs Institute (JBI) guidelines. MEDLINE, CINAHL, Scopus, Embase, and the Cochrane Library were searched, with publication language restricted to English and Italian. Primary studies and evidence syntheses addressing peristomal leakage were included. **Results**: Twenty-seven studies were included, most of which were primary observational studies conducted in Europe, North America, and the Nordic countries. Ileostomy was the most frequently investigated stoma type, followed by colostomy and urostomy. Across settings, peristomal leakage was highly prevalent, with most period or lifetime prevalence estimates exceeding 50%. Reported determinants clustered into anatomical, surgical, device-related, behavioral, care-related and psychosocial factors. Multiple tools were used, including leakage-specific and broader stoma questionnaires, but definitions and leakage grading were inconsistent. **Conclusions**: Peristomal leakage is a common, multifactorial, and largely preventable complication with substantial clinical, psychosocial and economic consequences. Clinical practice should prioritize early detection, validated assessment tools, patient education, specialized stoma nursing and structured follow-up. Future research should establish consensus definitions, robustly validate leakage-specific instruments, include under-represented regions and conduct high-quality economic evaluations to guide equitable, cost-effective care models.

## 1. Introduction

A stoma is a surgically created opening of the bowel or urinary tract that is placed on the abdominal wall, facilitating the diversion of fecal or urinary effluent [1,2]. The most prevalent types include colostomy and ileostomy [3]. Stoma formation is indicated for a broad range of conditions, including malignant diseases such as colorectal or bladder cancer, and benign disorders such as inflammatory bowel disease, diverticular disease, and traumatic abdominal or pelvic injuries [4]. In certain instances, a stoma may be temporarily established to protect distal anastomoses and allow healing, whereas in other situations, it serves as a permanent, life-preserving intervention [5].

The number of individuals living with a stoma is increasing worldwide. In the United States, an estimated 700,000 to 1,000,000 individuals currently live with a stoma, with approximately 120,000 new ostomies created annually [6]. In Europe, the prevalence is estimated at approximately 700,000 individuals [7]. However, determining exact numbers is difficult because of differences in reporting systems and the lack of standardized registries [7]. These data underscore the epidemiological and clinical significance of stoma care and the need to address the short- and long-term consequences associated with stoma creation.

Peristomal complications are highly prevalent and include peristomal skin complications (PSCs), parastomal hernia, prolapse, infections, bleeding, and granulomas [8,9]. Among these, peristomal leakage has been consistently reported as one of the most troublesome complications among patients, with implications for both clinical outcomes and psychosocial well-being [10].

Despite its importance, no single definition of peristomal leakage is universally accepted [11]. In the literature, leakage is often described according to two patterns: leakage under the baseplate (LUB), where effluent seeps beneath the adhesive barrier and remains in contact with the peristomal skin, and leakage outside the baseplate (LOB), where effluent escapes beyond the appliance and contaminates clothing or linen [11]. LUB is often insidious yet strongly associated with PSCs, whereas LOB has immediate and socially stigmatizing consequences [12], and some authors have defined leakage more broadly as the seeping of stomal output under the adhesive of the appliance and/or onto clothing or bedding [13]. From the patient’s perspective, leakage may also be understood as beginning when effluent first appears outside the skin barrier and reaches clothing, even if effluent has already been present beneath the barrier and in contact with the skin [7]. Distinguishing between LUB and LOB is fundamental in clinical practice because it helps clinicians better understand the likely mechanisms underlying leakage (i.e., whether it is predominantly mechanical/device-related or related to adhesion/seal failure) and guides more targeted management [14,15].

Peristomal leakage affects more than just local skin integrity. It is linked to limitations in daily activities, reduced quality of life, impaired social participation, and increased psychological distress [10,11,12,13]. Furthermore, leakage and associated peristomal skin complications (PSCs) lead to increased healthcare utilization, including unscheduled appliance changes, additional nursing and medical visits, and increased direct and indirect costs, substantially impacting both patients and healthcare systems [16]. Although various studies have investigated leakage, the evidence base remains fragmented and heterogeneous, with considerable variability in prevalence estimates, associated risk factors, and measurement approaches. To date, no comprehensive synthesis has been conducted to map the extent, nature, and characteristics of the available evidence on peristomal leakage. This scoping review aims to (i) determine the international prevalence of stoma leakage across regions and subpopulations; (ii) identify associated risk factors; and (iii) examine the measurement tools and strategies used to assess this complication.

## 2. Materials and Methods

A scoping review was conducted in accordance with the Joanna Briggs Institute (JBI) Manual for Evidence Synthesis guidelines [17] and the PRISMA-ScR checklist [18] to explore and map the extent of the literature on peristomal leakage. A scoping review enables the comprehensive mapping of existing evidence, the identification of knowledge gaps, and the clarification of key concepts to investigate and delineate the stoma leakage [19].

A structured a priori protocol was developed and followed rigorously throughout the review process; however, it was not prospectively registered or made publicly available in an online repository.

To explore and map the literature on peristomal leakage, the following research question was formulated: “What is the extent and nature of the existing evidence on peristomal leakage, specifically regarding its overall prevalence, prevalence within stoma subgroups, associated factors, and the instruments used to assess it?”

The Population/Concept/Context (PCC) framework, as recommended by the JBI guidelines [20], was used to guide the database searches and the development of search strategies and keywords (Table 1).

### 2.1. Eligibility Criteria

#### 2.1.1. Inclusion Criteria

This scoping review included records that addressed the research question and met the PCC framework criteria (Table 1). Eligible participants were adult and pediatric individuals with a colostomy, ileostomy or urostomy, regardless of the underlying disease leading to the stoma or gender. Primary qualitative and quantitative studies, as well as evidence syntheses, were considered for inclusion.

Regarding the concept of peristomal leakage, all records discussing this specific complication were included, irrespective of the type (e.g., leakage outside the baseplate) and without restriction to a specific phase of the condition (e.g., the immediate postoperative period or the long-term/chronic phase). The context was defined as broadly as possible to map the full body of literature on the topic, encompassing hospitals, clinics, and outpatient services, and was not limited by cultural or geographic setting.

#### 2.1.2. Exclusion Criteria

Records that did not address the PCC framework or the research question were excluded. Records pertaining to gray literature were excluded because they are typically difficult to analyze, systematically identify, and assess, and they are not peer-reviewed [21]. Records published in languages other than Italian or English were also excluded.

#### 2.1.3. Limits

Language filters (Italian and English) were applied to exclude records published in other languages. No time restrictions were applied to ensure that the search captured the entire body of literature on the topic.

### 2.2. Search Strategies

The study followed a three-step search strategy in line with JBI guidelines [20]. First, an initial, limited search was conducted in two key electronic databases relevant to the topic (MEDLINE via PubMed and CINAHL). The titles, abstracts, and indexing terms of the retrieved records were examined to identify relevant keywords and subject headings (Table S2). Second, these keywords and index terms were combined to develop a comprehensive search strategy that was executed in MEDLINE via PubMed, CINAHL, Scopus, Embase, and the Cochrane Library (Table S1). Third, the reference lists of the identified reports and articles were screened for additional sources. The search was conducted using the university and hospital library services, with support from an expert researcher. The second step of the search was conducted on 6 February 2025. Any documents not available in full text through the electronic databases were requested from the library service. If the library was unable to obtain them, the corresponding authors were contacted directly.

### 2.3. Document Selection

Study selection was reported in accordance with the 2020 PRISMA flow diagram [22]. Screening was conducted in two consecutive phases and managed using the RAYYAN web application, which also facilitated deduplication [23]. Initially, two independent reviewers (P.B. and E.D.N.) screened titles and abstracts to determine whether records met the predefined inclusion criteria. In the subsequent phase, the same reviewers evaluated the full texts of potentially relevant articles in detail. Disagreements were resolved through discussion; a third senior reviewer (A.P.) was available if consensus could not be reached, but this was not required. All references were organized, deduplicated, and stored in Zotero (version 7.0.29), followed by additional manual checking.

### 2.4. Data Extraction

Metadata were exported via Zotero (version 7.0.29) and manually verified after import. For the included studies, data were charted using a standardized extraction form based on the JBI Manual for Evidence Synthesis [17]. Given the focus of the review, the extraction template was expanded to capture information on the specific type of stoma and the corresponding prevalence of leakage, leakage-related complications, evaluation tools, outcomes and associated factors, and implications for practice.

This scoping review did not aim to undertake a formal appraisal of the methodological quality of the studies deemed eligible for inclusion, as this is not considered a core objective of scoping reviews in general [20].

### 2.5. Result Presentation

Given the scoping aim and the substantial heterogeneity across studies, evidence was synthesized using a narrative mapping approach supported by tables rather than quantitative pooling. Specifically, prevalence findings were reported as presented and organized by macro-area and by metric type (percent reporting ≥ 1 leakage episode within a stated recall period, episode frequency, or proxy indicators such as unplanned baseplate changes) and were considered non-comparable when leakage definitions/terminology, recall windows, populations or denominators, settings, or outcome metrics differed. Factors associated with leakage were charted verbatim and then grouped into anatomical/metabolic, technical or surgical, device-related, behavioral, care-related, and psychosocial domains, without comparing effect sizes because of variation in study designs and analytic reporting. Assessment methods were mapped by classifying instruments into validated leakage-specific tools, broader ostomy measures including leakage items, ad hoc questionnaires, and clinical/observational approaches, recognizing that tools often measured different constructs (frequency vs. impact vs. skin status). Cost evidence was summarized narratively and, where possible, distinguished between direct and indirect costs, but was not compared across settings due to sparse and inconsistently quantified reporting.

### 2.6. Stakeholder Engagement

No stakeholder engagement (e.g., patients, caregivers, stoma care nurses, or policy-makers) was conducted as part of this scoping review. This review aimed to map and summarize the published evidence; therefore, stakeholder consultation was not planned.

## 3. Results

The results have also been synthesized graphically in Figure S1.

### 3.1. Document Selection Process

The database search yielded a total of 14,358 records. After removal of deduplicated records, 8764 records remained for title and abstract screening.

At the end of this phase, 63 articles were retained for full-text assessment. Twelve full texts could not be retrieved, despite requests to the library services and attempts to contact the corresponding authors. Therefore, 51 articles were fully assessed.

**Figure 1:**
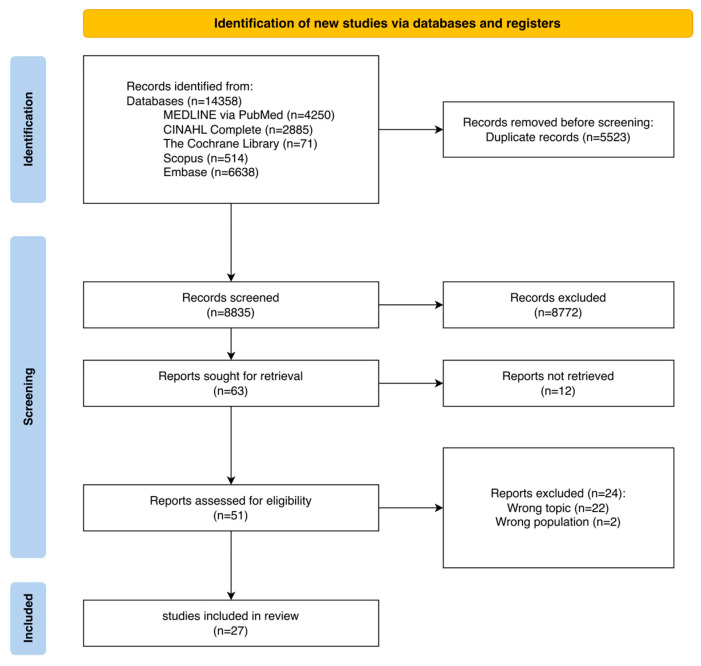
Document selection process (PRISMA 2020 flow diagram).

A total of 27 studies met the eligibility criteria and were included in the review. The study selection process is summarized in the PRISMA 2020 flow diagram (Figure 1) [22].

### 3.2. Characteristics of Included Studies

The 27 included studies were published between 1995 and 2025, with a marked increase in publications in the latter half of the period, beginning from early work by Bjerre et al. (1995) [24] to more recent trials [10]. The studies were conducted across four continents, predominantly in Europe (especially the United Kingdom and Denmark), with additional studies from North America, Asia, and Oceania. Most articles reported primary research, mainly observational studies (prospective, retrospective, cross-sectional, and modified Delphi designs), complemented by several interventional multicenter trials and one narrative review. Ileostomy was the most frequently investigated stoma type (92.6% of studies), followed by colostomy (88.9%) and urostomy (66.7%), with over half of the studies including all three types.

### 3.3. Prevalence of Peristomal Leakages

The prevalence of peristomal leakage showed a wide range of percentages, varying according to geographical area (Table 2 and Table 3).

#### 3.3.1. United Kingdom

Overall, leakage was very common: in one survey, 86% of participants reported at least one leakage episode in the previous month, and 69% had experienced effluent on their clothing in the previous year [39]. Another study found that 57% and 61% of patients experienced daytime and nighttime leakage, respectively, with most episodes occurring at least monthly [36]. Among patients undergoing corrective lipomodelling, the mean number of leakage episodes decreased from 2.25 to 0.5 episodes per 24 h at 6-month follow-up [33]. Device-focused studies showed that frequent leakage persisted despite conventional flanges; however, the introduction of new systems (e.g., Heylo or innovative appliances) halved baseplate changes due to fear of leakage and reduced leakage in up to 83% of users [10,34,35]. One study reported only the duration of the stoma (5–47 years) without quantifying leakage [37].

#### 3.3.2. Nordic Countries

Five studies from Denmark, Finland, Sweden and Norway reported heterogeneous, but still substantial, leakage patterns. In one trial the mean number of leakage episodes was 5.9 per week at baseline, decreasing to 1.8 after a new device was introduced [28]. Population-based surveys showed that approximately one-third of respondents had never experienced leakage, with the remaining two thirds reporting leakage episodes ranging from less than monthly to several times per week [12]. Another large study indicated that fewer than 20% of patients with a stoma had never experienced leakage, suggesting that the vast majority had experienced at least one leakage episode over time [30].

#### 3.3.3. United States

After stoma creation, 60% of patients reported leakage during follow-up [38]. In another study, 32% experienced weekly and 10% monthly leakage, with leakage episodes occurring slightly more often during the day (62%) than at night (60%) [31].

#### 3.3.4. East Asia

Two studies were conducted in East Asia. Among oncological patients, 6.7% developed leakage within three months of surgery [32]. In a randomized study evaluating a mobile app-based follow-up, the leakage rate was 1.75% in the intervention group compared with 16.1% in the control group, suggesting a potential preventive effect of structured remote support [15].

#### 3.3.5. Multinational Studies

Five multinational studies consistently reported a high frequency of peristomal leakage. Between 46.8% and 87% of participants reported at least one leakage episode within recall periods ranging from 7 days to 6 months [11,25,26,29]. Specifically, 46.8% experienced leakage in the week prior to the survey, 64.5% in the previous three months (mean 1.1 leakage episodes over the previous 14 days), and 76% in the previous six months, with higher prevalence among North American compared with European participants [26,27]. One study also showed that 76% experienced leakage under the baseplate at least monthly and 65% had effluent surpassing the flange edge at least once in the last year [27]. Collectively, these data indicate that peristomal leakage is a widespread problem across settings and countries, with only a minority of patients reporting no leakage episodes.

### 3.4. Prevalence of Peristomal Leakages by Stoma Type

Osborne et al. (2022) [25] reported that patients with ileostomies experience peristomal leakage more frequently than those with other stoma types. Although LeBlanc et al. (2019) [40] did not provide numerical estimates, they described leakage as a common event in patients with ileostomies and urostomies, particularly in hospital settings. In a study on urinary diversion, Bjerre et al. (1995) [24] observed more frequent, though manageable, leakage among patients with bladder substitution, whereas leakage was less common in those with ileal conduit urostomies. Down et al. (2021) [27] found that approximately 76% of participants had experienced leakage beneath the baseplate, and 65% had experienced leakage episodes in which effluent extended beyond the flange, contaminating clothing or linen. The same study reported that among patients with fecal stomas, only 2.9 of 10 baseplates were completely clean. In a mixed sample of individuals with ileostomy (62%) and colostomy (38%), de Fries Jensen et al. (2023) [15] showed that participants reporting lower concern about leakage experienced fewer leakage episodes (0.2–0.3 episodes, as reported). In an interventional study including colostomy, ileostomy and urostomy, González et al. (2021) [41] reported a baseline prevalence of 5.85 leakage episodes during the two weeks preceding study enrollment.

### 3.5. Associated Factors

Across the 27 included studies, multiple factors were associated with peristomal leakage. These associated factors are reported in Table 4.

#### 3.5.1. Anatomical and Metabolic Factors

The most frequently reported determinants were local anatomical characteristics of the peristomal area. Abdominal folds, scars, skin depressions or irregularities, stoma retraction, edema and concavities were repeatedly linked to poor adhesion and more complex appliance management [28,32,33,34,35,36,38,40,42,43]. Additional aspects included ileal conduit morphology in urostomies [24], the distance between the stoma and the umbilicus [32] and postoperative edema [44]. High BMI or obesity predisposed patients to peristomal irregularities and suboptimal seals [38,42]. Osborne et al. (2022) [25] further linked leakage with inadequate diet, poor appliance fit, skin complications and anatomical changes.

#### 3.5.2. Technical and Surgical Factors

Five studies identified surgical or technical contributors: absence of preoperative stoma-site marking [38], suboptimal stoma siting, position and shape [12,42,45], and emergency surgery [41].

#### 3.5.3. Device-Related Factors

Several studies focused on appliance characteristics. Inappropriate baseplate choice, such as using a flat baseplate instead of a convex one on irregular skin, was associated with higher leakage [28,41]. The use of standard, non-individualized devices and poor adhesion were also reported [42,43]. Two-piece systems showed a 78% lower probability of frequent leakage than one-piece pouches [31]. Prolonged wear beyond recommended times, particularly among North American patients, increased the risk of leakage [26]. Intensive use of accessories (seals, washers, rings, and pastes) was common in patients with leakage [25,36]. Inadequate cutting or sizing of the baseplate and poor adhesion of the device were additional contributors, and the specific appliance used influenced leakage management [14,27,45,46].

#### 3.5.4. Behavioral Factors

Behavioral aspects included the frequency and planning of appliance changes [25,26], technical difficulties in handling the device [42], and limited experience in stoma self-care [34]. Adherence, device availability, and appliance type were emphasized when evaluating leakage [14]. Patient skills and adherence to recommended techniques, as well as procedural errors in daily care, were also relevant [40,45]. Reduced mobility also emerged as an additional associated factor [43].

#### 3.5.5. Care-Related Factors

Pre- and postoperative information, unequal access to specialized stoma nursing and psychological support were identified as important care-related determinants [37]. Patients’ knowledge, skills and relationships with healthcare professionals were considered modifiable variables [12]. Inadequate education on the correct application and removal of appliances was directly linked to leakage [44].

#### 3.5.6. Psychosocial Factors

Psychosocial aspects included anxiety and fear of losing control of the appliance in public [11], and the need to consider patients’ emotions and perceived abilities when planning care [30]. Worry about leakage negatively affected mental health, quality of life and daily activities [14], and concerns about stoma visibility and effluent escape in social interactions were common [29]. Distress was greater among patients with ileal conduits and among those in lower social classes [24]. Female sex seems to be associated with higher incidence of leakage [31,38].

### 3.6. Assessment Tools

All 27 studies reported at least one method for assessing peristomal leakage, ranging from validated instruments to ad hoc questionnaires or clinical observation.

Hedegaard et al. (2020) [29] used the Ostomy-Q, which measures frequency and impact of leakage across four items (security, comfort, discretion, and social interaction). Similarly, Jeppesen et al. (2022) [28] applied the Ostomy Life Survey to define and quantify leakage. Nafees et al. (2018) [11] developed and validated the Ostomy Leak Impact Tool, which is organized into three domains (emotional impact, social functioning, and coping and control), and compared it with other validated scales. In a later study, Gunning et al. (2024) [14] employed two tools: the Ostomy Leak Impact (OLI), a 22-item scale with three domains (emotional impact, daily activities, coping and control), and the Ostomy Skin Tool 2.0 for grading peristomal skin lesions. The OLI was also used by Vendelbo et al. (2023) [37] and Osborne et al. (2022) [25], the latter again in combination with the Ostomy Skin Tool 2.0.

Several studies used broader or composite assessment batteries. For example, Pittman et al. (2014) [30] employed the Pittman Ostomy Complication Severity Index (OCSI), a 9-item Likert scale for clinical complications (including leakage), the Stoma Care Self-Efficacy Scale (14 items) and the Ostomy Adjustment Inventory-23 (OAI-23). Wang et al. (2024) [32] used the DET score for skin status, the AIS to assess appliance acceptance, and additional questionnaires on wear time, leakage frequency and skin complications. The validated Stoma-QoL questionnaire was used by Bonomi et al. (2016) [36] up to 6 months after surgery to assess aesthetic perception and the number of leaks. Earlier work by Bjerre et al. (1995) [24] relied on a 211-item questionnaire covering leakage distress, interruption of activities, body image, and information quality.

Other studies relied on study-specific questionnaires. Brady et al. (2025) [10] used Likert scales to measure concern about leakage, perceived security, and unplanned changes, and they applied the System Usability Scale (SUS) to assess pouch usability. Fellows et al. (2017) [26] used Likert-scale questionnaires examining leakage, perceived security, and unplanned baseplate changes. De Fries Jensen et al. (2023) [15] administered online questionnaires addressing leakage frequency, interaction with healthcare professionals, use of support products and related costs. Ratliff (2014) [38] developed a 24-item questionnaire covering demographics, clinical history, stoma type, leakage frequency and accessory use. Down et al. (2021) [27] used a standardized online questionnaire, within the Ostomy Life Study package, to capture leakage frequency, skin exposure, accessory use and concerns about device management. Redmond et al. (2009) [39] used a structured questionnaire developed by Theresa Parker and Associates; Evans and White (2020) [34] and Weidmann et al. (2014) [43] also used patient questionnaires to compare collection devices or record skin condition and appliance stability. Kruse and Størling (2015) [45] referred generically to multiple-choice questionnaires.

Some studies focused on clinical or observational tools. Ota et al. (2023) [31] collected data through a comprehensive stoma registration form including clinical variables, anatomical distances, scars and skin folds. González et al. (2021) [41] used the DET Ostomy Skin Tool and a Likert scale for patient-reported satisfaction. LeBlanc et al. (2019) [40] did not use formal scales but recommended the Peristomal Skin Assessment Guide (PSAG) for clinical evaluation. Burch (2013) [44] advocated systematic clinical observation rather than standardized tools. Brady et al. (2024) [33] and Weidmann et al. (2014) [43] used closed-ended questionnaires and nursing records to document peristomal skin status and appliance stability. Meisner and Balleby (2008) [42] similarly emphasized expert observational assessment and dialogue with the patient as the basis for timely, individualized interventions. Aibibula et al. (2022) [35] did not report any quantitative or standardized assessment tool.

### 3.7. Costs

Only five of the 27 studies addressed economic aspects [14,15,27,30,43], and cost information was generally sparse and not quantified in detail. No studies clearly differentiated direct from indirect costs or compared specific geographical areas. However, it can be inferred from these articles that poor management of leakage and devices increases direct costs, primarily through more frequent baseplate and pouch replacement, and indirect costs, including travel to specialized centers, additional clothing changes and the extra financial burden on healthcare systems and public services providing stoma supplies and support.

## 4. Discussion

This scoping review explores the high prevalence and clinical relevance of peristomal leakage across settings, with most studies reporting lifetime or period prevalence well above 50% [30,36]. Differences between high-income and low- and middle-income countries (LIMCs) appear to be linked less to whether leakage occurs than to how it is managed. In North America, patients report both more frequent leakage and greater anxiety about device stability than Europeans [26]. By contrast, a study conducted across four LMICs by Lapitan et al. (2024) [47] highlighted rationing and reuse of appliances, limited access to convexity, and chronic shortages of high-adhesion flanges. Together with the scarcity of specialized stoma nurses in these settings [28], these findings suggest that organizational and economic constraints may influence leakage management more than biological mechanisms [48]. Although evidence from LMICs remains scarce, limited access to advanced devices and specialized stoma care may increase the burden of leakage and create inequities in patient outcomes.

Across contexts, leakage emerges as a key precipitating factor for social isolation, aligning with Iovino’s distinction between disconnectedness and loneliness [49]. Fear of visible leakage or odor is nearly universal [25,26], leading to avoidance of social situations, and this fear is frequently reported as “severe” by those who experience daytime or nocturnal episodes [36]. Qualitative and quantitative data show a progression from anticipated stigma and activity restriction to enacted stigma and, over time, internalized shame and negative body image [11,31]. This progression also affects sexual and intimate relationships [24,50,51], which is mirrored by lower scores on the emotional and social domains of validated tools. Importantly, several interventions demonstrate that reducing leakage can partially reverse this trajectory: personalized appliances and tele-supported care improve acceptance, self-efficacy and Stoma-QoL scores within a few months [15,33,34].

The review also reinforces the multifactorial nature of leakage. Anatomical features (flat or retracted stoma, peristomal folds), surgical decisions (pre-operative marking, stoma height and site), device characteristics (one- versus two-piece systems, convex versus flat barriers) and patient behaviors (wear time, change routines) act in combination rather than in isolation [26,31,42,52,53]. This supports the conceptualization of leakage as the product of a complex system rather than a single “fault” in the device or the patient, indicating the need for integrated prevention strategies that combine optimal siting, tailored barriers, structured education and ongoing follow-up.

The setting of care emerged as an active component of this system. Studies from Scandinavia showed that leakage triggers additional consultations, accessory prescriptions, and higher workloads, and that better continuity with a specialized nurse is associated with fewer leakage episodes [12,54].

From a societal perspective, leakage translates into a substantial economic burden. It increases direct health-care costs leading to more frequent baseplate and pouch changes, greater use of accessories, extra visits and, in severe cases, emergency care and hospitalization [16,27,30,54]. Investing in more advanced barriers or digital alert systems may increase unit costs but can reduce peristomal skin complications and overall expenditure [55,56]. Direct non-health costs, including transport, laundry, clothing replacement and out-of-pocket purchase of support products, are particularly relevant in systems with limited reimbursement and can approach catastrophic levels for families in LMICs [47,57,58,59]. Indirect costs are less well quantified but likely substantial, given the reported impact of leakage on work, sleep, social participation and informal caregiving [27,30,60,61]. Together, these studies demonstrate the high economic consequences of leakage, including direct costs for appliances and indirect costs such as loss of productivity. Future studies should provide more detailed cost analyses to support health policy decisions.

Despite the proliferation of studies, critical methodological gaps remain. Only a minority of studies use validated tools specifically designed to capture leakage frequency, severity, and impact, such as the Ostomy Leak Impact Tool, OLI or Ostomy-Q [11,14,29,39], and even these instruments often focus on consequences rather than the standardized grading of leakage itself. Many surveys rely on ad hoc questionnaires or clinical observation, which limits comparability across studies. This highlights the urgent need for validated and internationally accepted tools to measure leakage consistently. The absence of a universally accepted definition and measurement framework for peristomal leakage makes the synthesis of prevalence estimates and the evaluation of interventions difficult. Moreover, the evidence base is heavily skewed toward high-income countries, with only a few contributions from LMICs, leaving major uncertainty about burden, risk factors and practical solutions in resource-constrained settings.

Finally, this review shares the intrinsic limitations of scoping methodologies. We mapped the breadth of available evidence without formally appraising study quality, and the marked heterogeneity in designs, populations, definitions and outcomes precluded meta-analysis and precise pooled estimates. However, by integrating clinical, psychosocial and economic findings, our synthesis highlights peristomal leakage as a multifaceted, largely preventable driver of morbidity and isolation. A further limitation is that, although an a priori protocol was developed, it was not prospectively registered. The absence of public registration may reduce transparency and limit readers’ ability to assess deviations from the original plan, potentially increasing the risk of reporting bias. We did not undertake a formal appraisal of the methodological quality or risk of bias of the included studies. While critical appraisal is not mandatory for scoping reviews, its absence prevents the weighting of findings by study rigor and limits the extent to which the robustness of the evidence supporting each reported association can be evaluated. Future research should prioritize consensus definitions, robust validation of leakage-specific scales, inclusion of under-represented regions, and high-quality economic evaluations to guide equitable and cost-effective models of care.

## 5. Conclusions

This scoping review is the first to provide a comprehensive overview of peristomal leakage, synthesizing data on prevalence, associated factors, assessment tools and costs. Leakage episodes are frequent across all types of stoma, particularly ileostomies, and arise from a combination of anatomical and technical issues, device characteristics, self-management behaviors, patient education and the patient’s lived experience. Despite their clinical relevance, accurate quantification is hampered by heterogeneous definitions and non-standardized assessment tools. Moreover, leakage is strongly associated with social isolation, stigma, and reduced quality of life. Addressing these psychosocial consequences should be considered a priority in both clinical practice and future research.

While scoping reviews do not support prescriptive clinical recommendations, the findings highlight areas that may warrant further attention and evaluation in care pathways, including early identification of leakage, the use of standardized/validated assessment approaches where feasible, therapeutic education with follow-up, and systematic outcome monitoring (e.g., through registries or routine data systems). Future studies should prioritize the development of internationally agreed definitions of peristomal leakage and the thorough validation of leakage-specific scales. Multicenter, cross-country collaborations are needed to generate robust epidemiological data and to include regions that are currently under-represented. At the same time, international consensus initiatives should be promoted to translate this evidence into shared, evidence-based clinical practice guidelines for the prevention, assessment and management of leakage. Finally, high-quality economic evaluations are required to inform fair, sustainable and cost-effective models of care for people living with a stoma.

## Figures and Tables

**Table 1:** PCC framework [20].

Framework Component		Description
P	Population	Individuals living with a stoma
C	Concept	Peristomal leakages
C	Context	Hospitals, community, clinics, outpatient services without any cultural or geographical limitations

**Table 2:** Leakage prevalence (proportion, %), reported with explicit construct and recall window.

Macro-Area	Study	Leakage Construct (as Reported)	Recall Window/Timepoint	Prevalence Metric (Unit)	Estimate
UK	Osborne et al., 2022 [25]	LUB (effluent under baseplate)	Past month	Period prevalence (%)	86%
		LOB (visible on clothing)	Past year	Period prevalence (%)	69%
Multi-national	Nafees et al., 2018 [11]	Leakage (not stratified by LUB/LOB)	Past 7 days	Period prevalence (%)	46.8%
Multi-national	de Fries Jensen et al., 2023 [15]	Leakage episode (not stratified by LUB/LOB)	Past 3 months	Period prevalence (≥1 episode, %)	64.5%
Multi-national	Fellows et al., 2017 [26]	Leakage (not stratified by LUB/LOB)	Past 6 months	Period prevalence (%)	76%
Multi-national	Down et al., 2021 [27]	LUB (under baseplate)	≥1 episode/month (frequency threshold)	Recurrent leakage prevalence (%)	76%
		LOB (beyond baseplate, soiling clothing/linen)	Past year	Period prevalence (%)	65%
North European countries	Jeppesen et al., 2022 [28]	Leakage (not stratified by LUB/LOB)	Ever/unspecified (reported as “never vs. ever”)	Ever prevalence (%)	≈80% (because <20% reported “never”)
Multi-national	Hedegaard et al., 2020 [29]	Leakage (definition suggests mainly LUB)	Device-change-based recall (reported over appliance changes; not directly time-based)	Prevalence (%)	87% (recall window not time-comparable)
USA	Pittman et al., 2014 [30]	Leakage during clinical follow-up (not LUB/LOB)	Post-operative follow-up (timepoint-based; not comparable to community recall windows)	Incidence/proportion (%)	60%
Asia	Ota et al., 2023 [31]	Leakage	Within 3 months post-op	Cumulative incidence (%)	6.7%
Asia	Wang et al., 2024 [32]	Leakage	Post-operative follow-up (trial; window not comparable with community recall windows)	Incidence/rate (%)	1.75–16.1% (by study arm)

**Table 3:** Leakage frequency and resource-use outcomes. Outcomes are not directly comparable to prevalence (%) and are thus presented separately with explicit units/time windows.

Macro-Area	Study	Outcome Type	Unit/Time Window	Estimate (as Reported)
UK	Brady et al., 2024 [33]	Resource use	Additional baseplate changes/week attributable to leakage	+2.47 changes/week
UK	Evans and White, 2020 [34]	Intervention effect	Reduction outcome (not a prevalence estimate)	83% reduction (as reported)
UK	Brady et al., 2025 [10]	Not quantifiable	Denominator not provided	No precise prevalence
UK	Aibibula et al., 2022 [35]	Qualitative finding	—	Leakage described as common; no prevalence
UK	Bonomi et al., 2016 [36]	Frequency	Mean leakage episodes/24 h	Mean 2.25/24 h
Northern European countries	Vendelbo et al., 2023 [37]	Frequency	Mean leakage episodes/week	Mean 5.9/week
Northern European countries	Indrebø et al., 2023 [12]	Frequency categories	Frequency distribution (time-based)	32.5% none; 37.5% once/month; 16.3% >1/month; 7.5% weekly; 6.3% >1/week
USA	Ratliff, 2014 [38]	Frequency categories	Weekly vs. monthly	32% weekly; 10% monthly
UK	Redmond et al., 2009 [39]	Time-of-day + frequency categories	Daytime/night-time; weekly/monthly	57% daytime; 61% night-time; 41% once/week; 33% once/month

**Table 4:** Factor-by-study summary table.

Factor Domain	Associated Factor (as Reported)	Direction	Studies Reporting the Factor	Level of Evidence (by Design)	Notes
Anatomical/metabolic	Peristomal irregularities (folds, scars, depressions/concavities), stoma retraction	↑ leakage/poor seal	Ota et al., 2023; Osborne et al., 2022 [25,31]	Observational; descriptive survey	Reported as determinants linked to adhesion and device management difficulties
Anatomical/metabolic	Post-operative edema	↑ leakage	Wang et al., 2024 [32]	Interventional/trial context	Mentioned as anatomical contributor
Anatomical/metabolic	High BMI/obesity	↑ leakage	Pittman et al., 2014 [30]	Observational/descriptive	Often linked to peristomal topography and fit issues
Anatomical/metabolic	Distance between stoma and umbilicus	↑ leakage (context-dependent)	Ota et al., 2023 [31]	Observational	Specific anatomical metric reported in the post-op study
Anatomical/metabolic	Inadequate diet/poor appliance fit/skin complications/anatomical changes	↑ leakage	Osborne et al., 2022 [25]	Descriptive survey	Cluster of correlated factors (may not represent independent predictors)
Technical/surgical	Absence of preoperative stoma-site marking	↑ leakage	Pittman et al., 2014 [30]	Observational	Perioperative process factor
Device-related	Flat vs. convex baseplate choice (mismatch to body profile)	↑ leakage when device not matched	Vendelbo et al., 2023 [37]	Interventional/trial context	Device selection was observed
Device-related	Two-piece vs. one-piece pouch system	↓ frequent leakage with two-piece (reported)	Ratliff, 2014 [38]	Descriptive survey	78% lower probability reported
Device-related	Prolonged wear time beyond recommendations	↑ leakage	de Fries Jensen et al., 2023 [15]	Descriptive survey	Particularly highlighted in North American participants
Device-related	Intensive use of accessories (rings/pastes/seals)	Associated with leakage (marker of complexity)	Down et al., 2021 [27]	Descriptive survey	
Behavioral	Frequency/planning of appliance changes	Associated with leakage patterns	Fellows et al., 2017; de Fries Jensen et al., 2023 [15,26]	Descriptive survey	
Behavioral	Technical difficulties handling the device/limited self-care experience	↑ leakage	Fellows et al., 2017 [26]	Descriptive survey	Skills and technique factors
Behavioral	Reduced mobility	↑ leakage	Not uniquely attributable from the excerpt	—	
Care-related	Unequal access to specialist stoma nursing and psychological support	↑ leakage/worse management	Brady et al., 2025 [10]	Mixed/descriptive	Often reported as contextual determinant
Care-related	Inadequate education on correct application/removal of appliances	↑ leakage	Wang et al., 2024 [32]	Interventional/trial context	Reported as care-related determinant
Psychosocial	Fear/anxiety about leakage in public	↑ perceived impact/possibly ↑ events	Nafees et al., 2018 [11]	Instrument development/validation	Strongly linked to emotional/social impact
Psychosocial	Worry about how leakage affects QoL, daily activities	↑ burden	Nafees et al., 2018 [11]	Instrument development/validation	Impact pathway rather than causal determinant
Psychosocial	Female sex	↑ leakage incidence (reported)	Pittman et al., 2014 [30]; Ratliff, 2014 [38]	Observational; descriptive survey	
Psychosocial	Lower concern about leakage linked to fewer episodes	↓ leakage episodes	de Fries Jensen et al., 2023 [15]	Descriptive survey	Directionality uncertain (episodes may drive concern)

↑ Increased risk or frequency of leakage (or related negative outcomes). ↓ Decreased risk or frequency of leakage.

## Data Availability

Data supporting the findings of this scoping review are available from the corresponding author upon reasonable request.

## Supplementary Materials

The following supporting information can be downloaded at https://www.mdpi.com/article/10.3390/nursrep16020046/s1: Table S1: Search strategies; Table S2: Keywords; Figure S1: Synthesis of results: evidence map of peristomal leakage.

## Public Involvement Statement

No patients or members of the public were involved in the design, conduct, reporting, or dissemination plans of this scoping review.

## Guidelines and Standards Statement

This scoping review manuscript was conducted in accordance with the JBI Manual for Evidence Synthesis for scoping reviews [17] and the PRISMA-ScR (Preferred Reporting Items for Systematic reviews and Meta-Analyses extension for Scoping Reviews) checklist [18].

## Abbreviations

The following abbreviations are used in this manuscript:

CINAHL	Cumulative Index of Nursing and Allied Health Literature
JBI	Joanna Briggs Institute
LMIC	Low–Middle-Income Country
LOB	Leakage Outside the Baseplate
LUB	Leakage Under the Baseplate
PRISMA	Preferred Reporting Items for Systematic reviews and Meta-Analyses
PSCs	Peristomal Skin Complications

## References

[1] Juma I.M., Qassim T., Saeed M.F., Qassim A., Al-Rawi S., Al-Salmi S., Salman M.T., Al-Saadi I., Almutawea A., Aljahmi E. (2023). Intestinal Stomas—Current Practice and Challenges: An Institutional Review. Euroasian J. Hepato-Gastroenterol..

[2] Tafuri A., Presicce F., Sebben M., Cattaneo F., Rizzetto R., Ferrara F., Bondurri A., Veltri M., Barbierato M., Multidisciplinary Italian Study group for STOmas (MISSTO) (2022). Surgical Management of Urinary Diversion and Stomas in Adults: Multidisciplinary Italian Panel Guidelines. Minerva Urol. Nephrol..

[3] Mulita F., Lotfollahzadeh S. (2025). Intestinal Stoma. StatPearls.

[4] Ambe P.C., Kurz N.R., Nitschke C., Odeh S.F., Möslein G., Zirngibl H. (2018). Intestinal Ostomy. Dtsch. Arztebl. Int..

[5] Gröne J. (2018). Stoma. Coloproctology.

[6] Maydick-Youngberg D. (2017). A Descriptive Study to Explore the Effect of Peristomal Skin Complications on Quality of Life of Adults With a Permanent Ostomy. Ostomy Wound Manag..

[7] Claessens I., Probert R., Tielemans C., Steen A., Nilsson C., Andersen B.D., Størling Z.M. (2015). The Ostomy Life Study: The Everyday Challenges Faced by People Living with a Stoma in a Snapshot. Gastrointest. Nurs..

[8] Babakhanlou R., Larkin K., Hita A.G., Stroh J., Yeung S.-C. (2022). Stoma-Related Complications and Emergencies. Int. J. Emerg. Med..

[9] Elnaim A.L., Wong M., Sagap I. (2024). Intestinal Stomas; Basics, Complications and Controversy: Systematic Review. Acad. Med. Surg..

[10] Brady R.R., Sheard D., Alty M., Vestergaard M., Boisen E.B., Ainsworth R., Hansen H.D., Ajslev T.A. (2025). Novel Digital Leakage Notification System Can Promote Better Stoma Management Routines: A Multicentre Clinical Trial. Br. J. Nurs..

[11] Nafees B., Størling Z.M., Hindsberger C., Lloyd A. (2018). The Ostomy Leak Impact Tool: Development and Validation of a New Patient-Reported Tool to Measure the Burden of Leakage in Ostomy Device Users. Health Qual. Life Outcomes.

[12] Indrebø K.L., Aasprang A., Olsen T.E., Andersen J.R. (2023). Factors Associated with Leakage in Patients with an Ostomy: A Cross-sectional Study. Nurs. Open.

[13] Porrett T., Nováková S., Schmitz K., Klimekova E., Aaes H. (2011). Leakage and Ostomy Appliances: Results from a Large-Scale, Open-Label Study in Clinical Practice. Gastrointest. Nurs..

[14] Gunning A., Virgin-Elliston T., Price C., Murray C., Ndlovu S., Summerson A. (2024). Development of a Leakage Impact Assessment for Patients with a Stoma, Who May Be Impacted by Leakage. Br. J. Nurs..

[15] De Fries Jensen L., Rolls N., Russell-Roberts P., Vestergaard M., Jensen M.L., Boisen E.B. (2023). Leakage of Stomal Effluent Outside the Baseplate Leads to Rise in Product Usage and Health Professional Interactions. Br. J. Nurs..

[16] Taneja C., Netsch D., Rolstad B.S., Inglese G., Lamerato L., Oster G. (2017). Clinical and Economic Burden of Peristomal Skin Complications in Patients With Recent Ostomies. J. Wound Ostomy Cont. Nurs..

[17] Aromataris E., Lockwood C., Porritt K., Pilla B., Jordan Z. (2024). JBI Manual for Evidence Synthesis.

[18] Tricco A.C., Lillie E., Zarin W., O’Brien K.K., Colquhoun H., Levac D., Moher D., Peters M.D.J., Horsley T., Weeks L. (2018). PRISMA Extension for Scoping Reviews (PRISMA-ScR): Checklist and Explanation. Ann. Intern. Med..

[19] Arksey H., O’Malley L. (2005). Scoping Studies: Towards a Methodological Framework. Int. J. Soc. Res. Methodol..

[20] Peters M.D., Godfrey C., McInerney P., Munn Z., Tricco A.C., Khalil H., Aromataris E., Lockwood C., Porritt K., Pilla B., Jordan Z. (2024). Scoping Reviews. JBI Manual for Evidence Synthesis.

[21] Mahood Q., Van Eerd D., Irvin E. (2014). Searching for Grey Literature for Systematic Reviews: Challenges and Benefits. Res. Synth. Methods.

[22] Page M.J., McKenzie J.E., Bossuyt P.M., Boutron I., Hoffmann T.C., Mulrow C.D., Shamseer L., Tetzlaff J.M., Akl E.A., Brennan S.E. (2021). The PRISMA 2020 Statement: An Updated Guideline for Reporting Systematic Reviews. BMJ.

[23] Ouzzani M., Hammady H., Fedorowicz Z., Elmagarmid A. (2016). Rayyan—A Web and Mobile App for Systematic Reviews. Syst. Rev..

[24] Bjerre B.D., Johansen C., Steven K. (1995). Health-related Quality of Life after Cystectomy: Bladder Substitution Compared with Ileal Conduit Diversion. A Quest. Survey. Br. J. Urol..

[25] Osborne W., White M., Aibibula M., Boisen E.B., Ainsworth R., Vestergaard M. (2022). Prevalence of Leakage and Its Negative Impact on Quality of Life in People Living with a Stoma in the UK. Br. J. Nurs..

[26] Fellows J., Forest Lalande L., Martins L., Steen A., Størling Z.M. (2017). Differences in Ostomy Pouch Seal Leakage Occurrences Between North American and European Residents. J. Wound Ostomy Cont. Nurs..

[27] Down G., Vestergaard M., Ajslev T.A., Boisen E.B., Nielsen L.F. (2021). Perception of Leakage: Data from the Ostomy Life Study 2019. Br. J. Nurs..

[28] Jeppesen P.B., Vestergaard M., Boisen E.B., Ajslev T.A. (2022). Impact of Stoma Leakage in Everyday Life: Data from the Ostomy Life Study 2019. Br. J. Nurs..

[29] Hedegaard C., Ajslev T., Zeeberg R., Hansen A. (2020). Leakage and Peristomal Skin Complications Influences User Comfort and Confidence and Are Associated with Reduced Quality of Life in People with a Stoma. World Counc. Enteros. Ther. J..

[30] Pittman J., Bakas T., Ellett M., Sloan R., Rawl S.M. (2014). Psychometric Evaluation of the Ostomy Complication Severity Index. J. Wound Ostomy Cont. Nurs..

[31] Ota E., Hiyoshi Y., Matsuura N., Ishikawa K., Fujinami F., Mukai T., Yamaguchi T., Nagasaki T., Akiyoshi T., Fukunaga Y. (2023). Standardization of Preoperative Stoma Site Marking and Its Utility for Preventing Stoma Leakage: A Retrospective Study of 519 Patients Who Underwent Laparoscopic/Robotic Rectal Cancer Surgery. Tech. Coloproctol..

[32] Wang J., Zhuang Z., Zhou J., Lu X., Chen S., Wang L., Chen Y. (2024). 3D Printing and Intelligent Technology Increase Convenience, Reliability, and Patient Acceptance of Ostomy Nursing: A Randomized Controlled Trial. Updates Surg..

[33] Brady R.R.W., Sheard D., Alty M., Vestergaard M., Boisen E.B., Ainsworth R., Hansen H.D., Ajslev T.A. (2024). Evaluating the Effect of a Novel Digital Ostomy Device on Leakage Incidents, Quality of Life, Mental Well-Being, and Patient Self-Care: An Interventional, Multicentre Clinical Trial. J. Clin. Med..

[34] Evans M., White P. (2020). Selecting Convexity to Improve and Maintain Peristomal Skin Integrity. Br. J. Nurs..

[35] Aibibula M., Burry G., Gagen H., Osborne W., Lewis H., Bramwell C., Pixley H., Cinque G. (2022). Gaining Consensus: The Challenges of Living with a Stoma and the Impact of Stoma Leakage. Br. J. Nurs..

[36] Bonomi R., Conway A.Z., Rapisarda I.F., Koulouglioti C., Sajid M.S., Betal D., Kalra L. (2016). Lipomodelling for the Management of Symptomatic Peristomal Contour Abnormalities: A Pilot and Feasibility Study. Color. Dis..

[37] Vendelbo G., Carlsson E., Tøndel L.T., Myller E., Sternhufvud C., Simonsen K.S., Munch P., Petersen B. (2023). Using Peristomal Body Profile Assessment to Improve Leakage-Related Quality of Life for Individuals with an Ostomy. Br. J. Nurs..

[38] Ratliff C.R. (2014). Factors Related to Ostomy Leakage in the Community Setting. J. Wound Ostomy Cont. Nurs..

[39] Redmond C., Cowin C., Parker T. (2009). The Experience of Faecal Leakage among Ileostomists. Br. J. Nurs..

[40] LeBlanc K., Whiteley I., McNichol L., Salvadalena G., Gray M. (2019). Peristomal Medical Adhesive-Related Skin Injury: Results of an International Consensus Meeting. J. Wound Ostomy Cont. Nurs..

[41] González E.R., Zurita C.D.P., Caballero G.A., Rodríguez A.H., Rodríguez E.Z., Blázquez E.G. (2021). Impact of Convex Ostomy Appliances on Leakage Frequency, Peristomal Skin Health and Stomal Protrusion. Gastrointest. Nurs..

[42] Meisner S., Balleby L. (2008). Peristomal Skin Complications. Semin. Colon Rectal Surg..

[43] Weidmann A.K., Al-Niaimi F., Lyon C.C. (2014). Correction of Skin Contour Defects in Leaking Stomas by Filler Injection: A Novel Approach for a Difficult Clinical Problem. Dermatol. Ther..

[44] Burch J. (2013). Stoma Complications: An Overview. Br. J. Community Nurs..

[45] Kruse T.M., Størling Z.M. (2015). Considering the Benefits of a New Stoma Appliance: A Clinical Trial. Br. J. Nurs..

[46] Burch J. (2014). Optimal Support Systems for Patients with Stomas—An Opinion Piece. NRR.

[47] Lapitan M.C.M., Sacdalan M.D.P., Lopez M.P.J., Cruz M.F.P., Msosa V.J., Ademuyiwa A.O., Alakaloko F.M., Jain R., Mahajan A., Michael V. (2024). Mixed-Methods Exploration of Challenges to Stoma Care for Ostomates in Four Low- and Middle-Income Countries: STomacARe For Improvement reSearcH (STARFISH) Study. J. Glob. Health Rep..

[48] Endeshaw D., Tesfaye T.D., Afewerk S., Adal O., Belayneh A.G., Bogale E.K., Yohannes S. (2024). Knowledge and Practice of Intestinal Ostomy Care Among Nurses in Bahir Dar City, Ethiopia: A Cross-Sectional Study. Sage Open Nurs..

[49] Iovino P., Vellone E., Cedrone N., Riegel B. (2023). A Middle-Range Theory of Social Isolation in Chronic Illness. Int. J. Environ. Res. Public Health.

[50] Lin S., Yin G., Chen L. (2023). The Sexuality Experience of Stoma Patients: A Meta-Ethnography of Qualitative Research. BMC Health Serv. Res..

[51] Paszyńska W., Zborowska K., Czajkowska M., Skrzypulec-Plinta V. (2023). Quality of Sex Life in Intestinal Stoma Patients—A Literature Review. Int. J. Environ. Res. Public Health.

[52] Hoeflok J., Salvadalena G., Pridham S., Droste W., McNichol L., Gray M. (2017). Use of Convexity in Ostomy Care: Results of an International Consensus Meeting. J. Wound Ostomy Cont. Nurs..

[53] Robertson I., Leung E., Hughes D., Spiers M., Donnelly L., Mackenzie I., Macdonald A. (2005). Prospective Analysis of Stoma-related Complications. Color. Dis..

[54] Persson E.I., Forsmark A., Scheffel G., Sternhufvud C., Carlsson E. (2025). Stoma Care Nurse Consultations Regarding Leakages and Peristomal Skin Complications During the First Year After Ostomy Creation: A Chart Review. Int. Wound J..

[55] Boisen E.B., Cawson M., De Fries Jensen L., Mealing S., Van Hest N. (2025). Cost-Effectiveness of a Digital Leakage Notification System (Heylo^TM^) for People with Ileostomies or Colostomies in the United Kingdom. PharmacoEconomics.

[56] Colwell J.C., Pittman J., Raizman R., Salvadalena G. (2018). A Randomized Controlled Trial Determining Variances in Ostomy Skin Conditions and the Economic Impact (ADVOCATE Trial). J. Wound Ostomy Cont. Nurs..

[57] Fautrel B., Boonen A., De Wit M., Grimm S., Joore M., Guillemin F. (2020). Cost Assessment of Health Interventions and Diseases. RMD Open.

[58] Jo C. (2014). Cost-of-Illness Studies: Concepts, Scopes, and Methods. Clin. Mol. Hepatol..

[59] Mohsin K.F., Ahsan M.N., Haider M.Z. (2024). Understanding Variation in Catastrophic Health Expenditure from Socio-Ecological Aspect: A Systematic Review. BMC Public Health.

[60] Nichols T. (2018). Health Utility, Social Interactivity, and Peristomal Skin Status: A Cross-Sectional Study. J. Wound Ostomy Cont. Nurs..

[61] Rolls N., Yssing C., Bøgelund M., Håkan-Bloch J., De Fries Jensen L. (2022). Utilities Associated with Stoma-Related Complications: Peristomal Skin Complications and Leakages. J. Med. Econ..

