# Supplementary material for: Stoma Leakage: Prevalence, Associated Factors, and Assessment Tools—A Scoping Review
Source: Nurs Rep. 2026 Jan 30;16(2):46. doi: 10.3390/nursrep16020046 (PMC12943642; doi:10.3390/nursrep16020046)

Review

Stoma Leakage: Prevalence, Associated Factors, and Assessment Tools – A Scoping Review

Table S1. Search strategies.

Database	Date	Search Strategy	Filters	Results
MEDLINE via PubMed	February 6, 2025	(((((((("Colostomy"[Mesh]) OR ("Ileostomy"[Mesh])) OR ("Surgical Stomas"[Mesh])) OR ("Enterostomy"[Mesh])) OR ("Ostomy"[Mesh])) OR ("Urinary Diversion"[Mesh])) OR ("Ureterostomy"[Mesh])) OR (("colostomy"[Title/Abstract] OR "ileostomy"[Title/Abstract] OR "urostomy"[Title/Abstract] OR "stoma"[Title/Abstract] OR "surgical stomas"[Title/Abstract] OR "enterostomy"[Title/Abstract] OR "ostom*"[Title/Abstract] OR "ostomate*"[Title/Abstract] OR "intestinal stoma"[Title/Abstract] OR "urinary diversion"[Title/Abstract] OR "cutaneous ureterostomy"[Title/Abstract] OR "ureterocutaneostomy"[Title/Abstract] OR "ureteroileocutaneostomy"[Title/Abstract] OR "urostomy"[Title/Abstract] OR "ureterostomy"[Title/Abstract] OR "stomata"[Title/Abstract] OR "pouching system"[Title/Abstract] OR "colostomy bag"[Title/Abstract] OR "ileostomy bag"[Title/Abstract] OR "urostomy bag"[Title/Abstract] OR "stoma bag"[Title/Abstract]))) AND (("soiling"[Title/Abstract] OR "leakage"[Title/Abstract] OR "leak"[Title/Abstract] OR "peristomal skin complication"[Title/Abstract] OR "seepage"[Title/Abstract]))	Language filters: English and Italian	4250
EBSCO CINAHL (Complete)	February 6, 2025	See the search strategy details		2885
Scopus	February 6, 2025	("colostomy" OR "ileostomy" OR "urostomy" OR "stoma" OR "surgical stoma" OR "enterostomy" OR "ostomy" OR "ostomate" OR "intestinal stoma" OR "urinary diversion" OR "cutaneous ureterostomy" OR "ureterocutaneostomy" OR "ureteroileocutaneostomy" OR "urostomy" OR "ureterostomy" OR "stomata" OR "pouching system" OR "colostomy bag" OR "ileostomy bag" OR "urostomy bag" OR "stoma bag") AND (	Language filters (English and Italian) Nursing Articles Reviews	514

		"soiling" OR "leakage" OR "leak" OR "peristomal skin complication" OR "seepage") AND (LIMIT-TO (LANGUAGE , "English") OR LIMIT-TO (LANGUAGE , "Italian")) AND (LIMIT-TO (DOCTYPE , "ar") OR LIMIT-TO (DOCTYPE , "re")) AND (LIMIT-TO (SUBJAREA , "NURS"))		
**Embase**	February 6, 2025	('colostomy'/exp OR 'colostomy' OR 'ileostomy'/exp OR 'ileostomy' OR 'stoma'/exp OR 'stoma' OR 'surgical stoma'/exp OR 'surgical stoma' OR 'enterostomy'/exp OR 'enterostomy' OR 'ostomy'/exp OR 'ostomy' OR 'ostomate' OR 'intestinal stoma'/exp OR 'intestinal stoma' OR 'urinary diversion'/exp OR 'urinary diversion' OR 'cutaneous ureterostomy'/exp OR 'cutaneous ureterostomy' OR 'ureterocutaneostomy'/exp OR 'ureterocutaneostomy' OR 'ureteroileocutaneostomy'/exp OR 'ureteroileocutaneostomy' OR 'urostomy'/exp OR 'urostomy' OR 'ureterostomy'/exp OR 'ureterostomy' OR 'stomata'/exp OR 'stomata' OR 'pouching system' OR 'colostomy bag'/exp OR 'colostomy bag' OR 'ileostomy bag'/exp OR 'ileostomy bag' OR 'urostomy bag'/exp OR 'urostomy bag' OR 'stoma bag'/exp OR 'stoma bag') AND ('soiling'/exp OR 'soiling' OR 'leakage'/exp OR 'leakage' OR 'leak'/exp OR 'leak' OR 'peristomal skin complication'/exp OR 'peristomal skin complication' OR 'seepage') AND ([english]/lim OR [italian]/lim) AND [humans]/lim AND [embase]/lim AND ('article'/it OR 'review'/it)	Language filters (English and Italian)<br>Humans<br>Articles<br>Reviews	6638
**The Cochrane Library**	February 6, 2025	See the search strategy details		71

Search History_Cumulative Index to Nursing and Allied Health Literature - CINAHL Complete [EBSCO]

Query	Limiters/Expanders	Results
S5	S4 AND S3	369
S4	S1 OR S2	
S3	TI (("leakage" OR "leak" OR "peristomal skin complication" OR "seepage")) OR AB (("leakage" OR "leak" OR "peristomal skin complication" OR "seepage"))	

S2	TI ((colostomy" OR "ileostomy" OR "urostomy" OR "stoma" OR "surgical stomas" OR "enterostomy" OR "ostom" OR "ostomate" OR "intestinal stoma" OR "urinary diversion" OR "cutaneous ureterostomy" OR "ureterocutaneostomy" OR "ureteroileocutaneostomy" OR "urostomy" OR "ureterostomy" OR "stomata" OR "pouching system" OR "colostomy bag" OR "ileostomy bag" OR "urostomy bag" OR "stoma bag")) OR AB ((colostomy" OR "ileostomy" OR "urostomy" OR "stoma" OR "surgical stomas" OR "enterostomy" OR "ostom*" OR "ostomate*" OR "intestinal stoma" OR "urinary diversion" OR "cutaneous ureterostomy" OR "ureterocutaneostomy" OR "ureteroileocutaneostomy" OR "urostomy" OR "ureterostomy" OR "stomata" OR "pouching system" OR "colostomy bag" OR "ileostomy bag" OR "urostomy bag" OR "stoma bag"))	
S1	(MM "Colostomy") OR (MM "Ileostomy") OR (MM "Surgical Stoma") OR (MM "Enterostomy+") OR (MM "Ostomy") OR (MM "Urinary Diversion") OR (MM "Ureterostomy")	

Search History_MEDLINE via PubMed

MEDLINE via MEDLINE				
Search number	**Query**	**Sort By**	**Filters**	**Results**
14	((((((("Colostomy"[Mesh]) OR ("Ileostomy"[Mesh])) OR ("Surgical Stomas"[Mesh])) OR ("Enterostomy"[Mesh])) OR ("Ostomy"[Mesh])) OR ("Urinary Diversion"[Mesh])) OR ("Ureterostomy"[Mesh])) OR (("colostomy"[Title/Abstract] OR "ileostomy"[Title/Abstract] OR "urostomy"[Title/Abstract] OR "stoma"[Title/Abstract] OR "surgical stomas"[Title/Abstract] OR "enterostomy"[Title/Abstract] OR "ostom*"[Title/Abstract] OR "ostomate*"[Title/Abstract] OR "intestinal stoma"[Title/Abstract] OR "urinary diversion"[Title/Abstract] OR "cutaneous ureterostomy"[Title/Abstract] OR "ureterocutaneostomy"[Title/Abstract] OR "ureteroileocutaneostomy"[Title/Abstract] OR "urostomy"[Title/Abstract] OR "ureterostomy"[Title/Abstract] OR "stomata"[Title/Abstract] OR "pouching system"[Title/Abstract] OR "colostomy bag"[Title/Abstract] OR "ileostomy bag"[Title/Abstract] OR "urostomy bag"[Title/Abstract] OR "stoma bag"[Title/Abstract]))) AND (("soiling"[Title/Abstract] OR "leakage"[Title/Abstract] OR "leak"[Title/Abstract] OR "peristomal skin complication"[Title/Abstract] OR "seepage"[Title/Abstract]))	Most Recent	English, Italian, Humans	4,264

13	((((((("Colostomy"[Mesh]) OR ("Ileostomy"[Mesh])) OR ("Surgical Stomas"[Mesh])) OR ("Enterostomy"[Mesh])) OR ("Ostomy"[Mesh])) OR ("Urinary Diversion"[Mesh])) OR ("Ureterostomy"[Mesh])) OR (("colostomy"[Title/Abstract] OR "ileostomy"[Title/Abstract] OR "urostomy"[Title/Abstract] OR "stoma"[Title/Abstract] OR "surgical stomas"[Title/Abstract] OR "enterostomy"[Title/Abstract] OR "ostom*"[Title/Abstract] OR "ostomate*"[Title/Abstract] OR "intestinal stoma"[Title/Abstract] OR "urinary diversion"[Title/Abstract] OR "cutaneous ureterostomy"[Title/Abstract] OR "ureterocutaneostomy"[Title/Abstract] OR "ureteroileocutaneostomy"[Title/Abstract] OR "urostomy"[Title/Abstract] OR "ureterostomy"[Title/Abstract] OR "stomata"[Title/Abstract] OR "pouching system"[Title/Abstract] OR "colostomy bag"[Title/Abstract] OR "ileostomy bag"[Title/Abstract] OR "urostomy bag"[Title/Abstract] OR "stoma bag"[Title/Abstract]))) AND (("soiling"[Title/Abstract] OR "leakage"[Title/Abstract] OR "leak"[Title/Abstract] OR "peristomal skin complication"[Title/Abstract] OR "seepage"[Title/Abstract]))	Most Recent	English, Italian	4,901
12	((((((("Colostomy"[Mesh]) OR ("Ileostomy"[Mesh])) OR ("Surgical Stomas"[Mesh])) OR ("Enterostomy"[Mesh])) OR ("Ostomy"[Mesh])) OR ("Urinary Diversion"[Mesh])) OR ("Ureterostomy"[Mesh])) OR (("colostomy"[Title/Abstract] OR "ileostomy"[Title/Abstract] OR "urostomy"[Title/Abstract] OR "stoma"[Title/Abstract] OR "surgical stomas"[Title/Abstract] OR "enterostomy"[Title/Abstract] OR "ostom*"[Title/Abstract] OR "ostomate*"[Title/Abstract] OR "intestinal stoma"[Title/Abstract] OR "urinary diversion"[Title/Abstract] OR "cutaneous ureterostomy"[Title/Abstract] OR "ureterocutaneostomy"[Title/Abstract] OR "ureteroileocutaneostomy"[Title/Abstract] OR "urostomy"[Title/Abstract] OR "ureterostomy"[Title/Abstract] OR "stomata"[Title/Abstract] OR "pouching system"[Title/Abstract] OR "colostomy bag"[Title/Abstract] OR "ileostomy bag"[Title/Abstract] OR "urostomy bag"[Title/Abstract] OR "stoma bag"[Title/Abstract]))) AND (("soiling"[Title/Abstract] OR "leakage"[Title/Abstract] OR "leak"[Title/Abstract] OR "peristomal skin complication"[Title/Abstract] OR "seepage"[Title/Abstract]))	Most Recent	English	4,863
11	((((((("Colostomy"[Mesh]) OR ("Ileostomy"[Mesh])) OR ("Surgical Stomas"[Mesh])) OR ("Enterostomy"[Mesh])) OR ("Ostomy"[Mesh])) OR ("Urinary Diversion"[Mesh])) OR ("Ureterostomy"[Mesh])) OR (("colostomy"[Title/Abstract] OR "ileostomy"[Title/Abstract] OR	Most Recent		5,394

	"urostomy"[Title/Abstract] OR "stoma"[Title/Abstract] OR "surgical stomas"[Title/Abstract] OR "enterostomy"[Title/Abstract] OR "ostom*"[Title/Abstract] OR "ostomate*"[Title/Abstract] OR "intestinal stoma"[Title/Abstract] OR "urinary diversion"[Title/Abstract] OR "cutaneous ureterostomy"[Title/Abstract] OR "ureterocutaneostomy"[Title/Abstract] OR "ureteroileocutaneostomy"[Title/Abstract] OR "urostomy"[Title/Abstract] OR "ureterostomy"[Title/Abstract] OR "stomata"[Title/Abstract] OR "pouching system"[Title/Abstract] OR "colostomy bag"[Title/Abstract] OR "ileostomy bag"[Title/Abstract] OR "urostomy bag"[Title/Abstract] OR "stoma bag"[Title/Abstract]))) AND (("soiling"[Title/Abstract] OR "leakage"[Title/Abstract] OR "leak"[Title/Abstract] OR "peristomal skin complication"[Title/Abstract] OR "seepage"[Title/Abstract]))			
**10**	((((((("Colostomy"[Mesh]) OR ("Ileostomy"[Mesh])) OR ("Surgical Stomas"[Mesh])) OR ("Enterostomy"[Mesh])) OR ("Ostomy"[Mesh])) OR ("Urinary Diversion"[Mesh])) OR ("Ureterostomy"[Mesh])) OR (("colostomy"[Title/Abstract] OR "ileostomy"[Title/Abstract] OR "urostomy"[Title/Abstract] OR "stoma"[Title/Abstract] OR "surgical stomas"[Title/Abstract] OR "enterostomy"[Title/Abstract] OR "ostom*"[Title/Abstract] OR "ostomate*"[Title/Abstract] OR "intestinal stoma"[Title/Abstract] OR "urinary diversion"[Title/Abstract] OR "cutaneous ureterostomy"[Title/Abstract] OR "ureterocutaneostomy"[Title/Abstract] OR "ureteroileocutaneostomy"[Title/Abstract] OR "urostomy"[Title/Abstract] OR "ureterostomy"[Title/Abstract] OR "stomata"[Title/Abstract] OR "pouching system"[Title/Abstract] OR "colostomy bag"[Title/Abstract] OR "ileostomy bag"[Title/Abstract] OR "urostomy bag"[Title/Abstract] OR "stoma bag"[Title/Abstract]))	Most Recent		89,461
9	("soiling"[Title/Abstract] OR "leakage"[Title/Abstract] OR "leak"[Title/Abstract] OR "peristomal skin complication"[Title/Abstract] OR "seepage"[Title/Abstract])	Most Recent		126,155
8	("colostomy"[Title/Abstract] OR "ileostomy"[Title/Abstract] OR "urostomy"[Title/Abstract] OR "stoma"[Title/Abstract] OR "surgical stomas"[Title/Abstract] OR "enterostomy"[Title/Abstract] OR "ostom*"[Title/Abstract] OR "ostomate*"[Title/Abstract] OR "intestinal stoma"[Title/Abstract] OR "urinary diversion"[Title/Abstract] OR "cutaneous ureterostomy"[Title/Abstract] OR	Most Recent		41,733

	"ureterocutaneostomy"[Title/Abstract] OR "ureteroileocutaneostomy"[Title/Abstract] OR "urostomy"[Title/Abstract] OR "ureterostomy"[Title/Abstract] OR "stomata"[Title/Abstract] OR "pouching system"[Title/Abstract] OR "colostomy bag"[Title/Abstract] OR "ileostomy bag"[Title/Abstract] OR "urostomy bag"[Title/Abstract] OR "stoma bag"[Title/Abstract])			
7	"Ureterostomy"[Mesh]	Most Recent		978
6	"Urinary Diversion"[Mesh]	Most Recent		16,528
5	"Ostomy"[Mesh]	Most Recent		55,825
4	"Enterostomy"[Mesh]	Most Recent		20,238
3	"Surgical Stomas"[Mesh]	Most Recent		2,66
2	"Ileostomy"[Mesh]	Most Recent		7,515
1	"Colostomy"[Mesh]	Most Recent		9,575

Search History_The Cochrane Library

Search number	Query	Sort By	Filters	Results
10	(#1 OR #2 OR #3 OR #4 OR #5 OR #6 OR #7 OR #8) AND #9	Relevancy	None	71
9	("soiling" OR "leakage" OR "leak" OR "peristomal skin complication" OR "seepage")		None	12248
8	("colostomy" OR "ileostomy" OR "urostomy" OR "stoma" OR "surgical stomas" OR "enterostomy" OR "ostomy" OR "ostomate" OR "intestinal stoma" OR "urinary diversion" OR "cutaneous ureterostomy" OR "ureterocutaneostomy" OR "ureteroileocutaneostomy" OR "urostomy" OR "ureterostomy" OR "stomata" OR "pouching system" OR "colostomy bag" OR "ileostomy bag" OR "urostomy bag" OR "stoma bag")		None	3915
7	MeSH descriptor: [Ureterostomy] explode all trees		None	12
6	MeSH descriptor: [Urinary Diversion] explode all trees		None	500
5	MeSH descriptor: [Ostomy] explode all trees		None	2202
4	MeSH descriptor: [Enterostomy] explode all trees		None	660
3	MeSH descriptor: [Surgical Stomas] explode all trees		None	161
2	MeSH descriptor: [Ileostomy] explode all trees		None	317
1	MeSH descriptor: [Colostomy] explode all trees		None	264

Table S2. Keywords

Free Keywords	MeSH Terms	CINAHL Subject Headings
**"colostomy"**	"Colostomy"[Mesh]	MH "Colostomy"
**"ileostomy"**	"Ileostomy"[Mesh]	MH "Ileostomy"
**"urostomy"**		
**"stoma"**		
**"surgical stomas"**	"Surgical Stomas"[Mesh]	MH "Surgical Stoma"
**"enterostomy"**	"Enterostomy"[Mesh]	MH "Enterostomy"
**"ostom*"**	"Ostomy"[Mesh]	MH "Ostomy"
**"ostomate*"**		
**"intestinal stoma"**		
**"urinary diversion"**	"Urinary Diversion"[Mesh]	MH "Urinary Diversion"
**"cutaneous ureterostomy"**		
**"ureterocutaneostomy"**		
**"ureteroileocutaneostomy"**		

**"urostomy"**		
**"ureterostomy"**	"Ureterostomy"[Mesh]	MH "Ureterostomy"
**"stomata"**		
**"pouching system"**		
**"colostomy bag"**		
**"ileostomy bag"**		
**"urostomy bag"**		
**"stoma bag"**		
**"soiling"**		
**"leakage"**		
**"leak"**		
**"pouching system leakage"**		
**"Leakage underneath the baseplate"**		
**"Leakage outside the baseplate"**		
**"peristomal skin complication"**		
**"seepage"**		
**"stoma bag leakage"**		

Figure S1: Synthesis of results: evidence map of peristomal leakage.

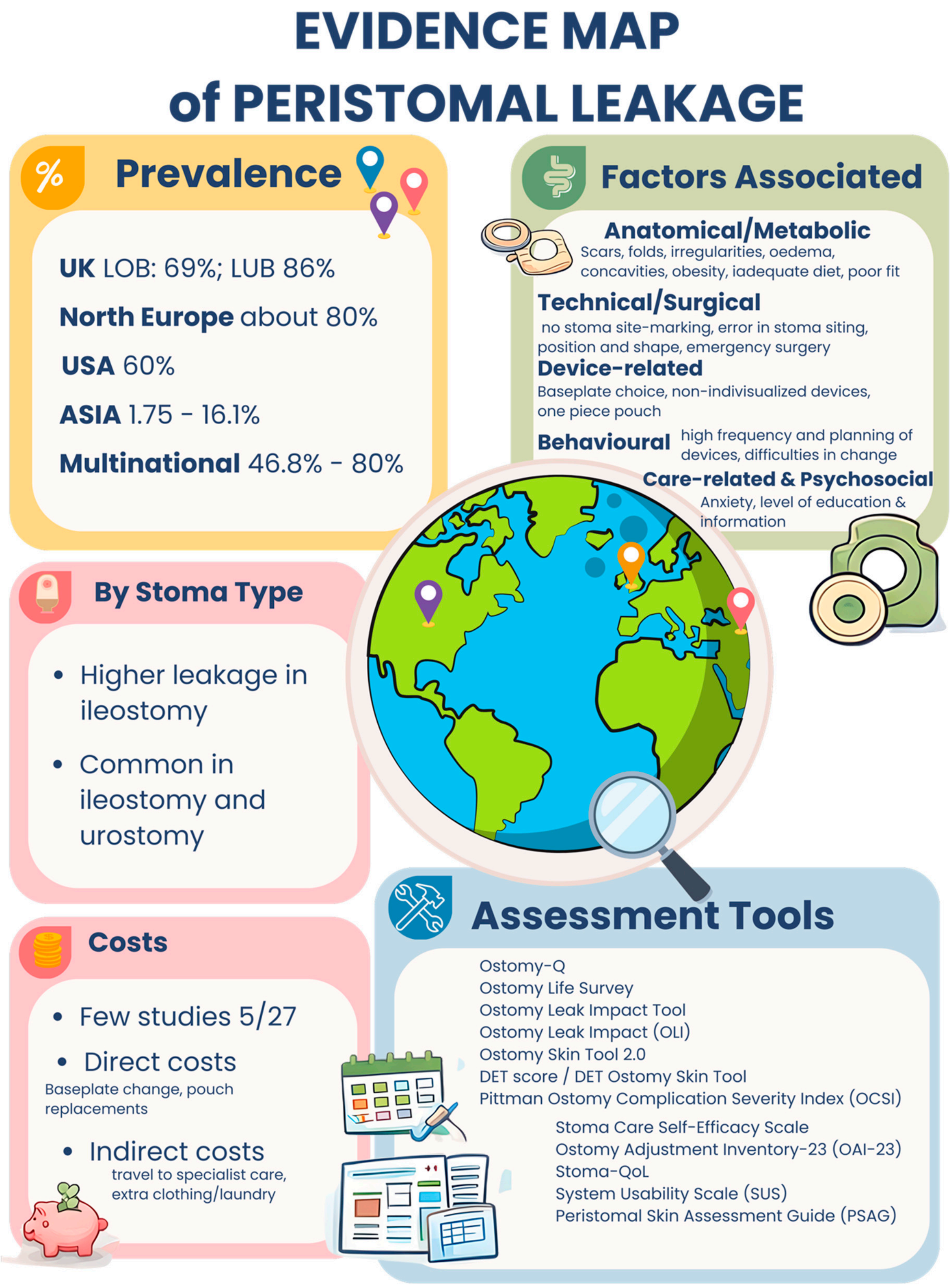

Supplement: Supplementary file 1 [file nursrep-16-00046-s001.zip › nursrep-4045148-supplementary.pdf]
